# Draft Genome Sequence for *Caulobacter* sp. Strain OR37, a Bacterium Tolerant to Heavy Metals

**DOI:** 10.1128/genomeA.00322-13

**Published:** 2013-06-06

**Authors:** Sagar M. Utturkar, Annette Bollmann, Ryann M. Brzoska, Dawn M. Klingeman, Slava E. Epstein, Anthony V. Palumbo, Steven D. Brown

**Affiliations:** Graduate School of Genome Science and Technology, University of Tennessee, Knoxville, Tennessee, USAa; Department of Biology, Northeastern University, Boston, Massachusetts, USAb; Department of Microbiology, Miami University, Oxford, Ohio, USAc; Biosciences Division, Oak Ridge National Laboratory, Oak Ridge, Tennessee, USAd

## Abstract

*Caulobacter* sp. strain OR37 belongs to the class *Alphaproteobacteria* and was isolated from subsurface sediments in Oak Ridge, TN. Strain OR37 is noteworthy due to its tolerance to high concentrations of heavy metals, such as uranium, nickel, cobalt, and cadmium, and we present its draft genome sequence here.

## GENOME ANNOUNCEMENT

*Caulobacter* spp. are Gram-negative bacteria that undergo asymmetric cell division and differentiation to produce two types of daughter cells: a motile swarmer cell and a division-compatible stalked cell (1, 2). Mobile swarmer cells are able to exploit new niches and eventually develop a tubular stalk that aids in surface attachment and nutrient uptake (3). The unique cell division pattern and ease of manipulation make *Caulobacter* spp. a simple single-celled model system to study cell cycle progression events. *Caulobacter* spp. are able to grow in low-nutrient environments, utilize dilute carbon sources, and degrade aromatic ring compounds (4) and have also been shown to possess surprisingly high tolerance to uranium (5, 6).

*Caulobacter* sp. strain OR37 was isolated from a site located at the Field Research Center (FRC) in Oak Ridge, TN (7), that is contaminated with radionuclides and heavy metals. Metagenomic, proteomic, and other functional approaches have been used to gain insights into the complex biogeochemical processes at contaminated sites (8, 9, 10, 11). Targeted efforts have aimed to bring formerly uncultivated organisms in culture and explore their functional roles, abilities, and adaptations to different environments (12, 13). We have recently sequenced the genome for *Microbacterium laevaniformans* strain OR221, a bacterium tolerant to metals, nitrate, and low pH that was also isolated from the FRC site (14). Strain OR37 was notable because it was tolerant to the highest tested concentrations of uranium (>200 µM), nickel (>500 µM), cobalt (>50 µM), and cadmium (>50 µM), while being inhibited only by the lowest pH and the highest concentration of nitrate (>250 mM) (7). We generated a draft genome sequence for *Caulobacter* sp. strain OR37 to gain insights into its physiology.

Draft genome sequence data for the strain OR37 were generated using a hybrid approach of 454 FLX (15) and Illumina MiSeq (16) technologies essentially as described previously (17). The 454 data consisted of 442,553 reads and generated 104,442,508 bp. Quality-trimmed Illumina data (CLC Genomics Workbench, version 5.5.1) consisted of 6,006,496 reads with an average length of 126 bp. Trimmed Illumina reads were assembled with CLC Genomics Workbench, and consensus sequences were distributed into 1.5-kbp overlapping fake reads using the fb_dice.pl script from the FragBlast module (http://www.clarkfrancis.com/codes/fb_dice.pl). The Newbler application (version 2.6, 454; Life Sciences) was used to generate a hybrid assembly from shredded Illumina reads and 454 data. The hybrid assembly consisted of 70 large (≥500 bp) contigs, with a total genome size of 4.4 Mb. The average contig size is 63,292 bp, with the largest contig being 604,995 bp, and the genome has an overall estimated G+C content of 67.7%.

The draft genome was annotated at the Joint Genome Institute (JGI) Integrated Microbial Genomes (IMG) database and comparative analysis system (18), which predicted 4,138 candidate protein-encoding gene models for *Caulobacter* sp. strain OR37. A number of heavy metal-translocating P-type ATPases, heavy metal efflux pumps, and metal ion efflux proteins were predicted, which might contribute toward its heavy metal resistance phenotype. This draft genome sequence will enable comparative genomics analysis and help to identify the genes responsible for heavy metal tolerance.

### Nucleotide sequence accession numbers. 

This Whole-Genome Shotgun project has been deposited at DDBJ/EMBL/GenBank under the accession no. APMP00000000. The version described in this paper is the first version, accession no. APMP00000000.
